# Hygienic assessment of fish handling practices along production and supply chain and its public health implications in Central Oromia, Ethiopia

**DOI:** 10.1038/s41598-022-17671-5

**Published:** 2022-08-17

**Authors:** Tesfaye D. Bedane, Getahun E. Agga, Fanta D. Gutema

**Affiliations:** 1 Department of Microbiology, Immunology and Veterinary Public Health, Addis Ababa University, P.O. Box 34, Bishoftu, Oromia Ethiopia; 2 Department of Veterinary Science, Salale University, P.O. Box 245, Fiche, Oromia Ethiopia; 3 Food Animal Environmental Systems Research Unit, Agricultural Research Service, U. S. Department of Agriculture, Bowling Green, KY 42101 USA; 4 Department of Occupational and Environmental Health, University of Iowa, Iowa City, IA 52246 USA

**Keywords:** Risk factors, Food microbiology

## Abstract

Fishborne diseases are among the major causes of morbidity and mortality worldwide. Contamination of the aquatic ecosystem and unhygienic handling practices along the fish supply chain can lead to a contaminated fish. Consumption of raw or under cooked fish and fish products is a major source of fishborne infections in humans. Despite reports of fish contamination with foodborne pathogens in Ethiopia, information regarding the hygienic status of fish handling practices is limited. We assessed fish hygienic handling practices at production sites and along the fish supply chain in three towns in east Shewa zone of Oromia. Data were collected using a semi-structured questionnaire interviews and personal observations. The study consisted of purposively selected respondents comprising of 50 fishermen, 10 retailers, 20 food establishments serving fish, and 120 consumers. Descriptive statistics and Chi-square test were used to present the proportion of various actors along the fish production and supply chain and to compare the proportions of observations among the different categories respectively. We observed that the lakes were accessible to animals and exposed to chemical and microbial contaminations through rainwater run-off. Fish were processed under unhygienic practices like washing of filleted fish with lake water, indiscriminate processing at unhygienic landing sites, use of a single knife for processing all fish with infrequent washing and with no disinfection in between. Majority (70%; n = 10) of the retailers and all the food establishments transported fish in vehicles with no cold chain facilities. Good hygienic practices we observed were the use of refrigerators for storage in all retailers and 70% (n = 20) of the food establishments; 30% of retailers used vehicles with a cold chain facility for the transportation of fish. Over three-fourths (77%; n = 120) of the consumers preferred consuming raw fish; 80% of them lacked the knowledge of fishborne diseases. The study revealed a wide range of unhygienic handling practices along fish production and supply chain; lack of infrastructure for post-harvest fish handling and processing, lack of appropriate transportation facilities and presence of knowledge gaps regarding fish borne diseases.

## Introduction

Fish is a food of excellent nutritional value providing high quality protein and unique long-chain polyunsaturated fatty acids and highly bioavailable essential micronutrients, vitamins D and B, and minerals. These compounds, often not readily available elsewhere in diets, have beneficial effects for adult health and child cognitive development^1^.

Production and supply of products or services involve various actors and networks with a wide range of activities that are required to bring a product or service from conception, through the different phases of production and supply chain to delivery to the final consumers^2^. In food industry, production and supply chain consist of actors that include producer, processor, wholesaler, exporter, importer, retailer, and consumer each with a unique role by adding a value to the products with an overall goal of supplying and accessing quality and safe food while generating maximum earnings from the business^3^. Within the context of fishery and aquaculture, fish production and supply chain start with collection of fish mostly from large water bodies such as lakes and oceans and end up delivering to consumer in markets far from thousands of miles. The major actors in the fish supply chain consists of a network of fishermen, retailers, distributors, transporters, storage facilities and suppliers that participate in the production, delivery, and sale of a product to the consumer^4,5^.

Long geographical distance that commonly exists between the fishermen and consumers makes the fish supply chain is a challenge in maintaining food quality and difficulty to tracing the pathways of the products when spoiled or poor-quality fish reaches the consumer^6^. In Ethiopia, fish production and distribution are operated both formally (e.g., organized cooperatives) and informally by individuals^7^. Alike in most developing countries, the fish supply chain in Ethiopia predominantly follows informal market system with limited supply chain management for fish quality and lack of information for price determination along the chain^8,9^. Under a predominantly informal marketing system which usually lack formal fish supply chain management and have no established system of traceability using either the traditional paper-based recording or the use of digital platforms such as blockchain technology^10,11^. In the absence of traceability, marketing and consumption of fish raise concerns of trust and transparency on the quality and safety^12,13^ and the price of the products which ultimately affects both fish consumers and sellers^14^.

Ethiopia has huge water bodies and wetland ecosystems that can support more than 200 fish species^15^ with an estimated average annual production potential of 94,500 metric tons of fish^16^. As a land locked country, fish production in Ethiopia entirely depends on inland water bodies such as lakes, reservoirs, and rivers. Approximately 75% of the total annual catch originates from the major lakes Tana, Dambel, Langano, Hawassa, Abaya and Chamo which are situated in the northwest and southern parts of the country. The remaining 25% of the total annual catch is harvested from minor lakes such as Beseka, Lugo, Hashengie, and Small Abaya, reservoirs such as Koka, Fincha-Amerti, Denbi, Melka-Wakena, Alwero, Tekeze, Gilgel Gibe I and major rivers^17^. For example, the Baro-Akobo basin drained by Baro, Akobo, Gilo and Alwero rivers, found in the Gambella region has an estimated fish production potential of 3,720 tons per year^18^.

Despite this huge fish production potential in Ethiopia, less attention has been given to the sector and the per capita fish consumption per year is very low (0.5 kg/year) due to poor integration of fish into the diet, lack of accessibility and limited supply^9^. If adequately utilized, fish production and marketing have different socio-economic significance in improving the livelihood of the community by ensuring food security, generating income, and providing self-employment opportunities for low-income groups particularly for young labor forces and rural communities dwelling near large waterbodies^9,19^.

The fishery sector in Ethiopia is hampered by several factors such as lack of adequate infrastructures at the fishing sites, lack of appropriate transportation facilities (including vehicles with thermostat), lack of harvesting and processing technologies and established supply chain management systems^9,20^. Consequently, various actors along fish production and supply chain can experience handling costs, financial loss due fish spoilage and more importantly consumers can lose trust and lack fast traceability information on the product quality. Thus, consumers can be exposed to contaminated fish due to lack of information related to the sources and hygienic conditions along the supply chain^21^. Besides, fish loss and fish waste due to improper handling have significant negative impacts on food-security, economy, and the environment by decreasing fish availability in the market, which may in turn increase fish prices and reduce the capacity of low-income consumers to access food. Moreover, if the quality of food deteriorates so badly that the fish must be sold at a lower price or even discarded to the environment as food waste, the livelihood of farmers and producers is adversely affected^22^. An investment at each step of the supply chain is required to improve fish handling by fully exploiting the resources using efficient solutions (e.g., the use of cold chain at each step) and establishing interlinkages among the actors to maintain fish quality and safety along the entire supply chain.

Fish contamination with chemical and microbial hazards can occur due to fish exposure to contaminated water with hazards from environmental sources linked with poor waste management such as from industries and livestock farms that pollutes the water resources^23^. Microbial contamination can occur at any point along the upstream supply chain during collection and processing, distribution, storage, marketing, and preparation if proper hygienic handling practices are not maintained^24–26^. Exposure to the microbial pathogens is a serious threat for consumer safety, especially when raw or undercooked fish is consumed^27^.

Bacterial diseases are considered the main cause of high mortalities and economic losses in fish industry worldwide^28^. *Mycobacterium* species, *Streptococcus iniae, Clostridium botulinum*, and *Vibrio vulnificus* are the major bacterial fish pathogens of public health concern. In addition, potential human pathogens such as *Salmonella typhi, Pseudomonas aeruginosa, Escherichia coli, Staphylococcus aureus,* and *Enterococcus faecalis* were isolated from edible fish and water samples^29^. The widespread distribution of these pathogens in the aquatic environments is mainly associated with potential contamination of the water bodies through run-off^30^. For instance, in Ethiopia the inland water bodies used as a natural source of fish receive many contaminant inflows from the nearby farmlands with fertilizers and untreated industrial wastes, municipal sewages, leaching from pit latrines and septic tanks^31^.

In Ethiopia, few studies have reported the occurrence of common fishborne pathogens. A review by^32^ showed that *Edwardsiella tarda, Salmonella*, *E. coli, S. aureus, Aeromonas* and *Vibrio* species are among the bacterial pathogens reported in fish in the country. The prevalence of these pathogens in fish varies considerably in different lakes across the country. For instance, *Salmonella* was reported at prevalence of 5.2% from Hayike^33^, 7.5% from Abaya and Chamo^34^and 30% from Tinike lakes^35^. Similarly, 1.5%^36^ and 2.3%^37^ prevalence of Shiga toxin producing *E. coli* O157:H7 were reported from fish samples tested at lake Hayike and Tekeze dam, and lake Hawassa, respectively.

Several bacterial pathogens cause infections and mortalities in fish and humans. People can be infected while handling infected fish on fish farms, at retail shops, or through ingestion of raw or inadequately cocked contaminated fish and/or fish products^38^. The widespread occurrence of these pathogens in fish along the fish supply chain could be due to lack of basic hygienic practices. Unhygienic handling practices lead to contamination of fish ready for human consumption with potentially foodborne pathogens; specially if fish is consumed raw or undercooked, it poses a significant public health risk^32^. Despite the availability of information on contamination of fish with common foodborne pathogens, data regarding fish hygienic handling practices along the production and supply chain is scarce in Ethiopia. The aim of this study was to assess the hygienic status of fish handling practices along production and supply chain in three towns of east Shewa zone of Oromia, Ethiopia that have natural lakes and potential reservoirs known to support fish production. Supply chain analysis is essential to closely examine the hygienic practices at each step along the production and supply chain to ensure fish quality and safety to increase consumer confidence. Information regarding the handling practices of fish at each step along the supply chain is critically needed to identify the critical control points and driving factors to devise preventive measures to ensure fish quality and safety. Moreover, it can pinpoint the extent of the problem to all stakeholders to initiate collective plans and actions to reduce associated costs and health risks and, optimize revenues to improve the livelihood of all actors relying on fishing business.

## Materials and methods

### Study area and study design

A cross-sectional study was conducted from December 2020 to June 2021 in Bishoftu, Koka and Batu towns of east Shewa zone of Oromia, Ethiopia. East Shewa zone is found in Central Oromia, connecting the western and eastern parts of Oromia. The zone has a total population of 1,356,342 within an area of 8,370.90 square kilometers with a population density of 162.03. While the majority (74.9%) of the population are mixed crop-livestock farmers or other rural dwellers, the remaining part of the population were urban inhabitants (25.1%) or few pastoralists (0.05%)^39^. Most of the fish population in Ethiopia is found in the rift-valley lakes, including a natural lake Dambel in Batu town, the crater lakes Babogaya and Hora-Arsedi in Bishoftu and reservoirs: Barbara or Koftu few kilo meters eastern to Bishoftu, and Koka reservoir near the town of Koka. Bishoftu, Koka and Batu towns are the three major towns in the east Shewa zone where fish is routinely harvested and consumed^40^.

Lake Babogaya is among the crater lakes of Bishoftu. Three fish species, namely *Oreochromis niloticus* (Nile tilapia), *Clarias gariepinus* (African Catfish) and *Tilapia zilli* were introduced into the lake by the Ministry of Agriculture to enhance the fishery resource of the lake. The most dominant species is *Oreochromis niloticus*; followed by *Clarias gariepinus*^41^. Lake Hora-Arsedi consists of only *O. niloticus* which was introduced around 1943^42^. Currently it supports a piscifauna, which is exclusively composed of Nile tilapia and *Tilapia zillii* although not much fishing is done except a few tons of fish caught for consumption by the town residents^43^. Barbara also known as (Koftu) is a man-made reservoir located few kilometers away from the eastern part of Bishoftu town. *Oreochromis niloticus* (Nile tilapia) is the only fish species inhabiting the reservoir. It was artificially introduced to the reservoir by the Bishoftu town office of livestock and fisheries with the objective of boosting fish production in the area^40^. Koka reservoir is the most important source of fish for small scale fisheries in general and riparian societies in particular^16^. It provides about 542 tons of fish annually. The currently existing commercially important species and their contribution to the total annual catch are common carp (*Cyprinus carpio* = 36%), catfish (*Clarias gariepinus* = 35%), tilapia (*Oreochromis niloticus* = 18%*)* and barbs (*Labeobarbus intermedius* = 11%)^44^.

The six indigenous fish species at Lake Dambel include *Barbus ethiopicus, Barbus paludinosus, Labeobarbus intermedius, Garra makiensis, Garra dembecha* and *oreochromis niloticus*^45^*.* In addition, three more exotic fish species such as T*ilapia zillii, Carassius carassius* and C*carassius auratu* were introduced into the lake with the objective of enhancing fish production, while the 4th exotic species, namely, *Clarias gariepinus,* is believed to have been slipped into the lake accidentally. Among others, tilapia (*Oreochromis niloticus* and *Tilapia zillii*), carp (*Carassius auratus* and *Carasiuscarrasius*) and catfish (*Clariasgariepinus*) are the most demanded and highly exploited type of fish species in the area^46^.

### Sample size and sampling

The sample size was estimated according to Shari^47^ who suggested a minimum required sample size of 50 participants for qualitative survey study. We collected data from a total of 200 selected respondents comprising 50 fishermen, 20 food establishment workers, 10 fish retailers and 120 consumers. Except for the later, the participants with more than one year of experience handling fish were purposively included in the study. Consumers were selected as convenience sample in the study based on their availability at the time of data collection at the restaurants or lakes, and their willingness to participate in the study. Questionnaire survey and direct observation were used to collect the data (supplementary file). All methods were performed in accordance with accepted ethics and regulatory requirements.

### Ethical clearance

The study was approved by Addis Ababa University ethical review committee (Ref No. VM/ERC/14/05/13/2021). Written informed consent was obtained from all study participants and privacy information are kept confidential to protect their business and identity.

### Data analysis

The data obtained was entered into an excel spreadsheet (Microsoft office^®^ excel 2007) and analyzed using Stata version 13.0 software (Stata Corp, College Station, TX)^48^. Descriptive statistics was used to calculate the frequency and proportion of various actors along the fish production supply chain involving handling, processing, transportation and storage practices, and consumption preferences. Chi-square test was used to compare the proportions of observations among the different categories. *P* < 0.05 was used for a statistical significance of an estimate.

## Results

### Demographic characteristics of the participants

Among a total of 200 respondents participated in the study, 90% of them were males. The mean age of the participants was 33.3 years (range 17–56 years) (Table 1).

**Table 1:** Gender and age distribution of respondents along fish production and supply chain in three towns of east Shewa zone of Oromia, Ethiopia.

Respondent category	Gender (number)		Mean Age (years)	Number (%) of respondents by town		
	Male	Female		Bishoftu	Koka	Batu
Fishermen (n = 50)	50	–	34.4	30 (60)	10 (20)	10 (20)
Fish retailers (n = 10)	7	3	33.8	1 (10)	1 (10)	8 (80)
Food establishment workers (n = 20)	1414	66	3939	7 (35)	6 (30)	7 (35)
Consumers (n = 120)	109	11	31.8	40 (33.3)	40(33.3)	40(33.3)

### Fish production and supply chain

**Figure 1:**
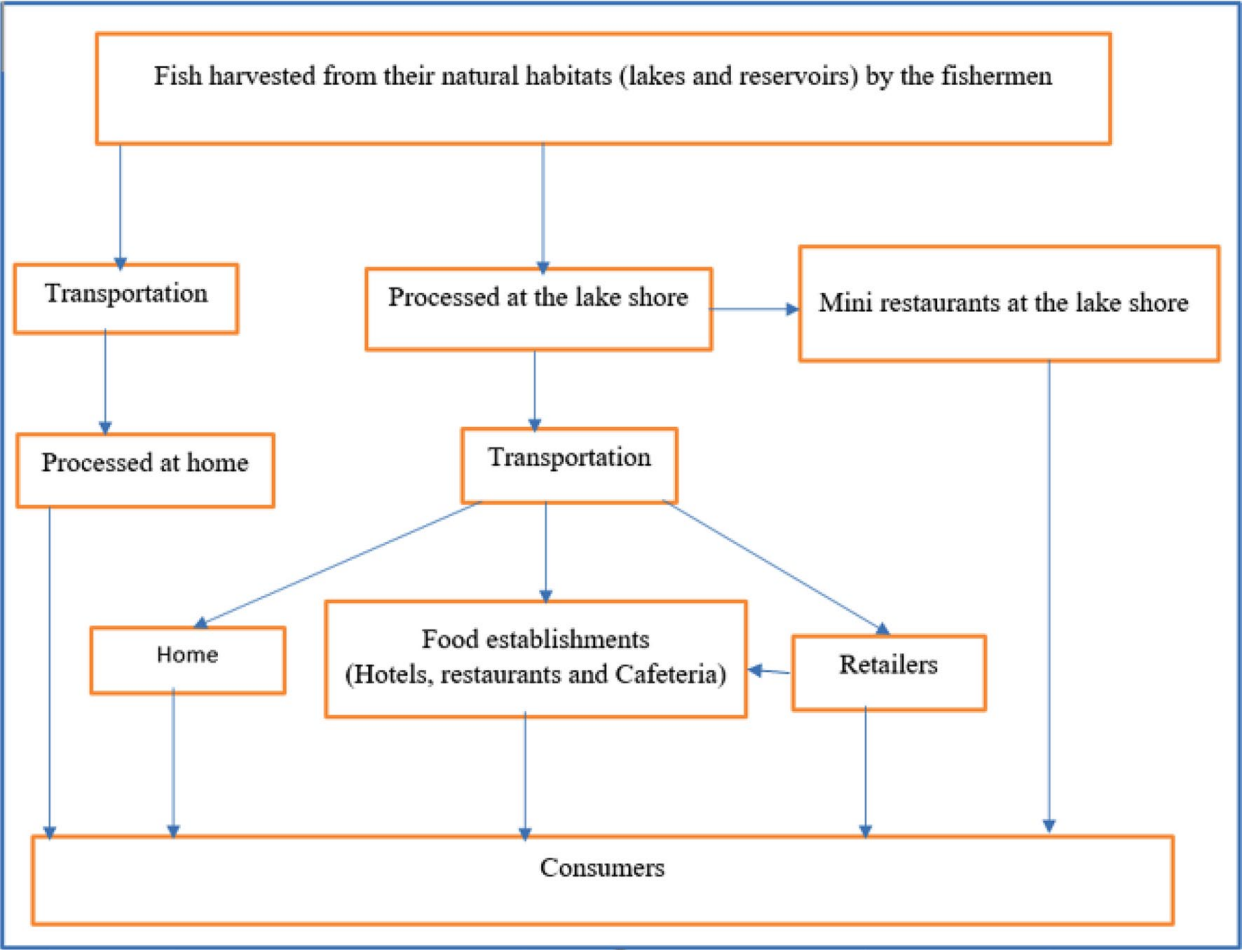
Conceptual flow diagram showing fish production and supply chain in east Shewa zone of Oromia, Ethiopia.

In the study area, fish is primarily harvested from freshwater lakes and reservoirs. The main actors in the supply chain were fishermen, fish retailers, food establishments and consumers. The fishermen take the lion’s share in harvesting and processing fish. All fishermen were male in this study (Table 1). Consumers can acquire fish through five different gateways each of which require their own standard of hygienic handling processes as shown in the fish production and supply chain conceptual framework depicted below (Fig. 1).

### Hygienic conditions at lakes and reservoirs

All the respondents reported that the lakes and reservoirs are accessible to animals, and most of them are watering points. They also indicated that an occasional open defecation by the people living and working around the lakes, and exposures of the water bodies to run-off water during the rainy season. We also observed animal feces, and cattle and cart horses roaming and grazing around the study lakes.

### Post-harvest handling and processing practices

It was observed that all the fishermen handled and processed fish under unhygienic conditions at the lake shore. All of them were performing the activity with no basic processing facilities such as clean and impervious processing room/area, clean/potable water, equipment washing and disinfection facility, hand washing facility, product handling facility. A single knife and the same cutting board were often used for the processing of all fish with infrequent washing and with no disinfection in between the processing. The fishermen visually inspect each fish for any external defects followed by manual incision to separate the head, tail, scale, and skin, gut removal and filleting. Filleted fish were washed using unclean water directly obtained from the lakes.

Table 2 summarizes fish processing, transportation, and handling practices by the fishermen. Majority (88%, n = 50) of the fishermen reported that their customers prefer processed fish at the shore compared to whole fish. Also, 84% of the fishermen reported selling the processed fish at the lake shore; and 64% of their customers were direct consumers. The most frequently used container by the fishermen for fish handling and transportation was a sack, and in some instances a plastic bag. They also used the same container (either sack or plastic bag) to transport a filleted fish for home consumption using a public transport with no cold chain.

**Table 2:** Fish processing, transportation, and handling practices by the fishermen (n = 50) in east Shewa zone, Oromia.

Variables	Category	% (number of observations)
Fish preference of customers	Whole fish	16 (8)
	Processed fish	84 (42)
Catching frequency	Every day	74 (37)
	Once per week	2 (1)
	Three times per week	24 (12)
Where to sale	At the lake shore	82 (41)
	At restaurants	12 (6)
	Used for personal consumption	6 (3)
Where to process	At the lake shore	88 (44)
	At home	12 (6)
Who will process	Fishermen	88 (44)
	Other processors	12 (6)
Fish customers	Consumers	64 (32)
	Retailers	14 (7)
	Hotels/restaurants	16 (8)
	Used for personal consumption	6 (3)
Total		100 (50)

Only 30% of retailers transported their fish using vehicles equipped with cold chain facilities. Overwhelmingly 70% of them used either ordinary trucks or a public transport with no cold chain using sacks and/or crate containers. All retailers stored fish in a refrigerator for a maximum duration of 7 days, but they never checked the storage temperature.

Similarly, none of the food establishments used a cold chain facility for fish transportation; and 70% of them stored fish in a refrigerator for a maximum duration of 24 h. However, none of them checked the storage temperature. The remaining 30% of the food establishments have reported that no fish will remain from the daily supply, and they did not store fish.

### Fish consumption

Among the 120 consumers who completed the questionnaires, 13.3% of them preferred raw fish only, while the majority (63.3%) of them preferred either raw or heated/fried fish. Thus, about 77% of the consumers had the preference of consuming raw fish. There was statistically significant difference in the fish consumption preference based on gender and study sites (*P* < 0.05) (Table 3). The majority (81.8%, n = 11) of female consumers preferred consumption of heat-treated fish. However, there was no difference in the fish consumption preferences among the consumers based on age, religion, educational status, occupation, or spatial location (study site) (*P* > 0.05).

**Table 3:** Fish consumption preferences of different categories of the respondents (n = 120).

Variables	Category	% Consumption preference (Number of observations)			Chi-square, *P*-value
		Raw	Heat treated	No preference	
Study site	Bishoftu (n = 40)	15 (6)	40 (16)	45 (18)	12.4, 0.015
	Koka (n = 40)	10 (4)	10 (4)	80 (32)	
	Dambel (n = 40)	15 (6)	20 (8)	65 (26)	
Gender	Male (n = 109)	14.68 (16)	18.35 (20)	66.97 (73)	16.8, < 0.001
	Female (n = 11)	0.00 (0)	72.73 (8)	27.27 (3)	
Education	No formal education (n = 6)	0.00 (0)	33.33 (2)	66.67 (4)	9.6, 0.291
	Adult education (n = 5)	20 (1)	40 (2)	40 (2)	
	Primary (n = 63)	9.52 (6)	15.87 (10)	76.60 (47)	
	Secondary (n = 41)	19.51 (8)	29.27 (12)	51.22 (21)	
	Higher (n = 5)	20 (1)	40 (2)	40 (2)	
Religion	Orthodox (n = 112)	13.39 (15)	25 (28)	61.61 (69)	2.8, 0.247
	Protestant (n = 8)	12.50 (1)	0.00 (0)	87.50 (7)	
Occupation	Bajaj driver (n = 3)	0.00 (0)	66.67 (2)	33.33 (1)	38.75, 0.347
	Cart driver (n = 2)	0.00 (0)	50 (1)	50 (1)	
	Daily worker (n = 5)	0.00 (0)	20 (1)	80 (4)	
	Farmer (n = 10)	10 (1)	20 (2)	70 (7)	
	Fish cooker (n = 8)	0.00 (0)	50 (4)	50 (4)	
	Fish retailer (n = 4)	25 (1)	25 (1)	50 (2)	
	Fish processor (n = 6)	33.33 (2)	16.67 (1)	50 (3)	
	Fish restaurant owner (n = 1)	0.00 (0)	100 (1)	0.00 (0)	
	Fish dealer (n = 1)	0.00 (0)	0.00 (0)	100 (1)	
	Fishermen (n = 22)	4.55 (1)	13.64 (3)	81.82 (18)	
	Hotel owner (n = 3)	0.00 (0)	33.33 (1)	66.67 (2)	
	Isuzu driver (n = 1)	0.00 (0)	0.00 (0)	100 (1)	
	Public servant (n = 16)	18.75 (3)	25 (4)	56.25 (9)	
	Student (n = 4)	25 (1)	0.00 (0)	75 (3)	
	Taxi driver (n = 2)	50 (1)	50 (1)	0.00 (0)	
	Driver assistant (n = 2)	0.00 (0)	50 (1)	50 (1)	
	No fish dealer (n = 13)	38.46 (5)	23.08 (3)	38.46 (5)	
	Waiter (n = 10)	10 (1)	0.00 (0)	90 (9)	
	Shoe polisher (n = 7)	0.00 (0)	28.57 (2)	71.43 (5)	

Majority (83.3%) of the consumers had no knowledge about the significance of avoiding cross contamination between heat treated fish and raw fish including other raw foods (Table 3). Furthermore, 80% of them had no knowledge about fishborne zoonotic diseases. Consumers believed that the use of spices like red pepper, commonly consumed with a raw fish, and consumption of local alcoholic beverage called katikala would avoid the risk of acquiring fishborne illnesses.

## Discussion

In this study, we assessed the hygienic status of fish handling practices at the production sites and using a combination of questionnaire survey and direct observation approaches to identify the fish supply chain and to qualitatively measure the hygienic conditions along the supply chain. The approaches we used are essential in generating relevant information needed as inputs in food quality assessment model. However, we did not investigate the prevalence and concentration of pathogenic microorganisms and examine the effects of unhygienic handling practices on fish microbial quality and safety of fish along the supply. Further comprehensive food safety risk assessment that consists of hazard identification at each critical point, hazard characterization (dose response relationship), human exposure assessment to the hazards via consumption of contaminated fish and risk characterization (e.g., illness per exposed people) is required to quantify the probable risk and identify the risk mitigation measures to implement efficient and feasible fish safety management system along the supply chain^49,50^. Nonetheless, our study revealed significant basic hygienic gaps in the fish handling practices that can be targeted for interventions. Considering the inherent differences in the scope and outcomes of the different food safety assessment approaches, we discussed the convergences and divergences of the findings from the perspectives of national and global sets of standards and regulatory requirements. Specifically, the major findings at each critical points in the fish supply chain were discussed in view of Ethiopian national proclamations (Ethiopian Public Health Proclamation No. 200/2000 and Ethiopian Food and Medicine Administration Proclamation No. 1112/2019), and internationally recommended hygienic standards and regulatory requirements (FAO, WHO and Codex Alimentarius Commission, CAC) for hygienic handling of fish to prevent fish contamination to ensure fish safety and to reduce financial loss due to fish spoilage.

The free access of cattle to the freshwater lakes and reservoirs, the exposure of the water bodies to contaminated water run-off and open defecation by people in the area are potential factors that can lead to the contamination of the natural habitats for fish production. We also observed the use of contaminated water for fillet washing which is contrary to FAO and WHO code of practice for fish and fishery products^51^.The code affirms the availability of adequate supply of clean sea water or potable water for washing fish prior to filleting or cutting, fillets after filleting, skinning, or trimming and filleting equipment and utensils. The codex general principle of food hygiene also declares the control of water quality to minimize the risk of potential biological, chemical, and physical hazards^52^. The Ethiopian public health proclamation no. 200/2000 prohibits the supply of any food which is unhygienic, contaminated, and unwholesome, or does not meet the standards of food safety and quality^53^. Therefore, the indiscriminate contamination of freshwater lakes and reservoirs from which fish is massively produced and consumed coupled with the use of potentially contaminated water for fillet washing at the production sites are important areas of concern for public health where interventions can be targeted.

Fecal contamination that may contain pathogens from animals and people particularly as the result of the observed open defecation can reach the lakes through run-off water. This contradicts the general principle of food hygiene^52^, which states that the control of fecal contamination minimizes the potential for contamination with many foodborne pathogens such as *Salmonella*, *Campylobacter*, *Yersinia* and pathogenic strains of *E. coli*. Most importantly, contaminated water run-off may carry antimicrobial residues and resistant pathogens that can play an important role in the dissemination of antimicrobial resistance and transmission of such resistant pathogens among fish, animals and humans^50^. According to FAO and WHO^51^, bilateral code of practice for fish and fishery products, contamination of fish depends on the environment and the bacteriological quality of the water from which it is harvested. Once the aquatic environment is contaminated with a zoonotic pathogen, it is highly probable for the aquatic fauna including fish to acquire the pathogen and become a potential threat for fish consumers^54^. Thus, limiting the access of animals to the water bodies, diverting the direction of water run-off against the natural habitat of fish, and circumventing the open defecation by providing access to public restrooms around the beaches and at the shores are the most likely intervention strategies. Our study found significant breaches in the basic hygienic practices where the environment is likely contaminated by animal and human feces.

It was observed that, fish was washed with lake water having a potential risk of contamination with pathogenic microbes. According to FAO^54^, cross-contamination and contact with contaminated water may lead to dissemination of microbial contaminants into other fresh or heat-treated fishery products. Thus, due to unhygienic landing sites and unrestricted cross contamination of the processed products, the situation worsens the safety of consumers.

Our study indicated that fish processing was practiced by all fishermen at unhygienic landing sites at the lake shores that can result in the contamination of the fish. This contrasts with the recommendations of FAO, which states unhygienic post-harvest handling and processing procedures deteriorate the safety of consumers^54^. Fish requiring gutting on arrival at the processing facility should be gutted efficiently, without undue delay and with care to avoid contamination^55^. A study by Huss et al.^56^ also showed that contamination of fishery products is primarily due to unhygienic processing or poor water quality. Training of fishermen and infrastructural development at the landing sites are important interventions to promote hygienic fish processing in the area.

Our observation of the use of the same knife and one cutting board for processing all fish without disinfection of equipment shows risky practices that contribute to contamination and cross contamination of fish during production. The European commission health and consumer protection directorate requires that equipment and premises approved for slaughtering, carcass preparation and meat cutting to be carefully cleaned and disinfected several times during and at the end of the working day, and before being re-used, to prevent contamination by pathogenic microbes or other microbes associated with meat spoilage. In addition, the European union meat hygiene directives require that during the production process the temperature of water used to decontaminate hand-held tools (knives, hooks, saws) should be maintained at 82 °C or higher^57^. However, these basic facilities were not present in the present study area and intensive training of the fishermen on the concepts of contamination, cross contamination and an overall hygienic production and processing of fish are significant tools to reduce fishborne illnesses and to increase the shelf life of the fish.

Fish transportation and storage practices are not in line with the required standards. All food establishments did not have refrigerated vehicles for transportation; and only 30% of the retailers shipped fresh filleted fish using vehicles equipped with cold chain facilities. The use of unhygienic sacks or plastic bags as a packaging material is also a common practice. However, according to FAO^54^ fish is one of an exceptionally perishable product and post-harvest handling, processing and transportation channels should be performed under hygienic and cool temperature conditions to prevent microbial contamination and fish loss due to spoilage. The transportation and storage methods of fish should also be environmentally friendly, and assure the safety of consumers^58^. The recent recommendations of FAO and WHO^59^ also declares that fresh fish should be transported using clean and suitable vehicles at a temperature closer to 0 °C, preferably in containers with dry freezer bags instead of ice while frozen products should be maintained at – 18 °C or below. It also obliges the use of clean, sound, and durable packaging materials and maintaining adequate protection against contamination from dust, exposure to higher temperatures and the drying effects of the sun or wind^59^. The Ethiopian Food and Medicine Administration and Control Proclamation No. 1112/2019 also enforces the use of clean and suitable packaging material, adhering to the national and international quality and safety standards^60^.

The Codex Code of practice for fish and fishery products requires that fish should be maintained at the chilling temperature of 4 °C for short time storage and freezing temperature of ≤ − 18 °C for long time storage with regular temperature monitoring^51^. However, all retailers and 70% of the food establishments in the study area stored fish in a refrigerator for a maximum duration of 24 h and 7 days respectively, but none of them had known the storage temperature at which the fish were kept. Interestingly, 30% of the food establishments did not store fish; they supply consumers with freshly produced fish though the way it has been harvested, processed, and transported is still under sub hygienic conditions. This entails that fish retailers and food establishments should make every effort to store fish at required temperature to avoid fish spoilage and to ensure consumer safety.

The present study showed that consumption of raw fish is popular in the study area where about 77% of the respondents had the preference of consuming raw fish. In addition, 80% of them did not know the potential risk of fishborne pathogens associated with the consumption of contaminated fish. FAO^54^ indicated that food poisoning including consumption of contaminated fish, occurs because of eating raw or undercooked products or cooked products that have been cross-contaminated. Several studies elsewhere showed the linkage of the raw fish consumption with fishborne diseases. For instance, a study by Kalimuddin et al.^61^ showed that 283 infections due to *Streptococcus agalactiae* was linked to the consumption of raw fish in Singapore. Austin et al.^38^ also reported the presence of several bacterial pathogens of public health concern, causing serious morbidity and mortality in humans, through consumption of raw or inadequately cocked contaminated fish and/or fishery products. As stated above Shiga toxin producing *E. coli* O157:H7 was reported from fish examined at various lakes in Ethiopia^36,37^ implying the potential risk of consuming raw or inadequately coked fish or fishery products. However, the Ethiopian food safety regulations almost exclusively target commercial markets while most people consume what they produce or buy from the local informal markets^62^. Therefore, adoption, establishment and maintenance of operational safety standard protocols will ensure the safety of fish and fishery products to protect consumer health^22,58^.

Although the number of female participants included in the questionnaire survey was small, the study identified a significant gender difference with respect to raw fish consumption. In addition, in the study area, most of the fish handling practices (processing, transportation and storage) are managed by the male counterparts under unhygienic conditions and without adhering to the national or international safety standards. This is contrary to the FAO and WHO, code of conduct for responsible fisheries^59^, which declares a remarkable importance of naturally occurring food safety hazards in the environment from which fish are harvested. To that end, risks to consumer health associated with fish and fishery products obtained from unpolluted marine environments are low provided that the products are handled in line with the principles of good manufacturing practices. However, it would be increased when fish is mishandled along the value chain^59^. The report of Austin et al.^38^ also showed the potential for public health risk of handling infected fish on fish farms or in retailer shops. It implies that in men the risk of occupational exposure to fishborne pathogens (hazards) is more likely. So, women are less likely to be exposed to fishborne pathogens as compared to men and also by avoiding raw fish consumption.

Taken all together, the study indicates that the identified unhygienic handling practices that could be due to less stringent food safety regulation practices in Ethiopia calls for an urgent timely intervention to establish and maintain effective national fish quality and safety plans to ensure public safety. More importantly, consumption of a heat-treated fish should be promoted as effective risk mitigation strategy through public education to prevent risk of acquiring fishborne zoonotic diseases.

Moreover, the informal fish marketing system that dominates in Ethiopia should be regulated for implementation of effective supply chain management system. To reduce the perceived risk associated with food consumption and to increase the consumers trust, many countries have introduced Food Traceability System such as HACCP (Hazard Analysis and Critical Control Points), blockchain technology and other electronic systems; which can provide an information platform for all the supply chain members^12,63–65^. Blockchain technology in particular is a digital ledger platform which is becoming an important tool in different industrial production and Agri business in managing food supply chain by providing secure and immutable information^12,14^. Blockchain is a digital, decentralized, and distributed ledger in which transactions are logged and added in chronological order with the goal of creating permanent and tamper-proof records^33,66,67^. One of the most critical aspects in the use of blockchain applications is related to monitoring social and environmental conditions to control and avoid the occurrence of health and safety problems^68^. It provides the ability to track food such as fish and fish products through all stages of production, processing, and distribution to signal quality in the food supply chain^12,21^.

Under the current Ethiopian context, it is more prudent to transform the prevailing informal fish production and marketing system by strengthening the existing fish producer and supplier cooperatives to a large-scale commercial enterprise, establishing well networked formal marketing system, and regulating the market dynamics and improving infrastructural development. These will be foundational to evaluate the application of the digital technologies such as blockchain and adopt the technology in future studies for sustainable management of fish production and supply chain by addressing perishability of fish, demand–supply mismatch, unfair prices, and quality related data to satisfy the demands of intervening actors across the supply chain to encourage transparency and accountability in the entire value chain in Ethiopia^5^.

## Conclusion

Our study revealed a wide range of unhygienic handling practices along fish production and supply chain such as the use of contaminated lake water for fillet washing; the use of a single knife and cutting board to process multiple fish with infrequent cleaning and without any disinfection between processing; and the use of unhygienic fish containers and packaging materials. Furthermore, the study identified lack of infrastructure for post-harvest fish handling and processing, evidence of conspicuous cross-contamination during processing, lack of appropriate transportation facility and lack of adequate knowledge about fish borne diseases linked with the consumption of raw fish. The unhygienic handling practices and the raw fish consumption preference of the consumer imply the potential risk of fishborne diseases in the area necessitating timely interventions. Infrastructural development, establishing standard fish processing plant/house with basic facilities, provision of food safety training to all actors along the production supply chain and the use of easy to clean containers and cold chain facilities for transportation of fish are critically needed to ensure fish safety. Further studies should aim at investigating the role of the unhygienic handling practices, microbial quality of fish and other risk factors for the occurrence and contamination level of fish with foodborne pathogens to identify critical control points along the production and supply chain at which future interventions will be targeted. In addition to investigation into the intrinsic fish qualities such as changes in colour, aroma, flavor and texture of fish due to spoilage, examining the extrinsic factors such as method of production and processing, price determination, duration of storage, context and appropriateness (purchase/consumption occasion), origin and geographic distance between production and market, and consumption patterns are required for evidence based evaluation and decision for fish quality to enhance consumers’ confidence and acceptance and promote willingness to choose and pay for better quality. Moreover, to curb the immense economic losses and implications of public concerns on fish quality and safety emanating from lack of information on how the fish is produced and marketed along the value chain, evaluation, and adoption of various food supply management systems such as blockchain technology are critically required.

## Supplementary Information

Supplementary Information.

## Data Availability

All the generated data during this study were incorporated to the manuscript.

## Abbreviations

CAC: Codex Alimentarius Commission
CSA: Central Statistics Authority
ECHCPD: European Commission Health and Consumer Protection Directorate
EFNG: Ethiopian Federal Negarit Gazeta
ESZOLF: East Shewa zone Office of Livestock and Fisheries
FAO: Food and Agriculture Organization
HLPE: High Level Panel of Experts
WHO: World Health Organization
