# Supplementary material for: Hygienic assessment of fish handling practices along production and supply chain and its public health implications in Central Oromia, Ethiopia
Source: Sci Rep. 2022 Aug 17;12:13910. doi: 10.1038/s41598-022-17671-5 (PMC9385613; doi:10.1038/s41598-022-17671-5)
Supplement: Supplementary file 1 — Supplementary Information. [file 41598_2022_17671_MOESM1_ESM.docx]

**Supplementary file**

**Questionnaires to assess hygienic status of fish handling practices along production and supply chain in Central Oromia, Ethiopia**

1. **Fishermen**

**Part -1: Basic Information**

**Date: _____________**

**Study Site: ____________________**

**Respondent ID**: _______________

**Sex:**

1. Male
2. Female

**Age**: _______ (yrs)

**Educational level:**

1. Illiterate
2. Adult education
3. 1^0^ education
4. 2^0^ education
5. 3^0^ education

**Part-2: Specific Questions**

1. For how long have you been engaged in fishing? _______ (yrs)
2. How often do you catch a fish per week?
3. Once
4. Twice
5. Three times
6. Every day
7. How many fishes you catch per fishing day? ________
8. For what purpose do you catch a fish?
9. For personal consumption
10. For income generation
11. If you catch a fish for income generation, where do you sell your fishes?
12. At the lake shore
13. At retailer shops
14. In the open market
15. Hotel
16. Who are your major customers?
17. Consumers
18. Retailer shop owners
19. Hotels
20. Fish traders
21. What is the price of a kilo of raw fish meat in your town /village?

a) During fasting _______ (ETB)

b) During non-fasting ________ (ETB)

1. What about the fish preference of your customers (whole fish or processed)?
2. Whole fish
3. Processed fish
4. If your customers prefer a processed fish, who will process the fishes?
5. I will process by myself
6. I will give to other people who will process
7. Whether you process or others process, where does the fish processing takes place?
8. At home
9. At the lake shore
10. If you process the fishes at home or supply to the market or retailer shops, how do you transport them?
11. In a sack
12. In a crate
13. Hanging on hand without container
14. On a cart or Bajaj without container
15. Do you use an ice box or any other cold chain facility for fish transportation?
16. Yes
17. No
18. Do cattle graze around the lake from where you are catching fish?
19. Yes
20. No
21. Do people living or working around the lake have the trend of open defecation?
22. Yes B. No
23. Do the lake directly accessible to run-off water?
24. Yes
25. No

**Thank You So Much!**

**B- Fish meat consumers**

**Part -1: Basic Information**

**Date: _____________**

**Study Site: ____________________**

**Respondent ID**: ________________

**Sex:**

1. Male
2. Female

**Age**: _______ (yrs)

**Educational level:**

1. Illiterate
2. Adult education
3. 1^0^ education
4. 2^0^ education
5. 3^0^ education

**Part-2: Specific Questions**

1. Do you like a fish meat?
2. Yes
3. No
4. If yes, how do you prefer to consume?
5. Raw
6. Roasted /Cooked
7. Both raw and roasted /cooked
8. How often do you consume?
9. Every day
10. Every 3 days
11. Weekly
12. Every 2 weeks
13. Every 3 weeks
14. Monthly
15. Do you know any disease of fish which can be transmitted from fish to humans?
16. Yes
17. No
18. If yes, what are the major means of transmission?
19. Unhygienic handling of fish /fish meat
20. Consumption of raw and /or under cooked fish meat
21. Poor storage condition of fish /fish meat
22. Poor personal and environmental hygiene
23. Lack of awareness of acquiring disease through fish consumption
24. Other (specify)______________________________________
25. Have you ever encountered any diarrheic disease related to fish meat consumption?
26. Yes
27. No
28. Is there any one from your family who has such history?
29. Yes B. No
30. If yes, which group of your family had been encountered the case?
31. Child
32. Adult
33. Pregnant woman
34. Elder
35. Have you or your family member visited a health center or hospital due to such a case?
36. Yes
37. No
38. If yes, what was its response to treatment?
39. Rewarding B. Refractory

**Thank You So Much!**

**C- Fish meat retailers**

**Part -1: Basic Information**

**Date: _____________**

**Study Site: ____________________**

**Respondent ID**: ________________

**Sex:**

1. Male
2. Female

**Age**: _______ (yrs)

**Educational level:**

A. Illiterate

1. Adult education
2. 1^0^ education
3. 2^0^ education
4. 3^0^ education

**Part-2: Specific Questions**

1. Who are the major fish or fish meat suppliers for your retailer shop?
2. Fishermen
3. Fish traders
4. Which lakes are the major sources of your fish?
5. Bishoftu lakes
6. Koka reservoir
7. Lake Ziway
8. Arbaminch lakes
9. Both Arbaminch lakes and lake Ziway
10. Other, Specify__________________
11. How do you or your suppliers transport a whole fish or fish meat from source lakes to your retailer shop?
12. In a sack, using a public transport
13. In a sack, using an Isuzu vehicle
14. In a crate, using a public transport
15. In a crate, using an Isuzu vehicle
16. In a vehicle having a cold chain facility
17. At your retailer shop, where do you store your fish or the fish meat until it will be sold?
18. In a crate, on the ground
19. In a sack, on the ground
20. In a refrigerator
21. Without a sack or crate, on the ground
22. Other, Specify______________
23. If you use a refrigerator, what is your optimum storage temperature? _________ ^0^C
24. For how long do you store? ________(days)
25. Who are your major customers?
26. Consumers
27. Hotels
28. Fish traders
29. Other, specify_____________
30. What is the price of a whole raw fish in your retailer shop?
31. During fasting ___________(ETB)
32. During non-fasting ___________(ETB)
33. What about the price of a kilo of raw fish meat?
34. During fasting ___________(ETB)
35. During non-fasting ___________(ETB)
36. How many ETB does a roasted whole fish costs?
37. During fasting __________(ETB)
38. During non-fasting _________(ETB)

**Thank You So Much!**
